# Evaluation of vancomycin pharmacokinetics in patients with augmented renal clearances: A randomized clinical trial

**DOI:** 10.3389/fphar.2022.1041152

**Published:** 2022-11-11

**Authors:** Zahra Sahraei, Ali Saffaei, Ilad Alavi Darazam, Jamshid Salamzadeh, Minoosh Shabani, Shervin Shokouhi, Najmeh Sarvmeili, Mohammadreza Hajiesmaeili, Masood Zangi

**Affiliations:** ^1^ Department of Clinical Pharmacy, School of Pharmacy, Shahid Beheshti University of Medical Sciences, Tehran, Iran; ^2^ Critical Care Quality Improvement Research Center, Loghman Hakim Hospital, Shahid Beheshti University of Medical Sciences, Tehran, Iran; ^3^ Infectious Diseases and Tropical Medicine Research Center, Shahid Beheshti University of Medical Sciences, Tehran, Iran

**Keywords:** area under the curve, augmented renal clearances, hyperfiltration, kidney, pharmacokinetic, vancomycin

## Abstract

**Purpose:** Vancomycin is a narrow therapeutic window glycopeptide antibiotic that acts against Gram-positive bacteria. As it is renally eliminated, therapeutic drug monitoring is recommended for vancomycin, especially in case of kidney function alteration. Augmented renal clearance (ARC), defined as a creatinine clearance of more than 130 ml/min, is a risk factor for sub-therapeutic concentrations of vancomycin. This study aimed to evaluate the vancomycin pharmacokinetics following the administration of two different regimens in ARC patients.

**Methods:** A randomized clinical trial (IRCT20180802040665N1) was conducted on patients in need of vancomycin therapy. Eight hours of urine was collected and 56 patients divided into two groups with creatinine clearance of more than 130 ml/min were included in the study. The first group received 15 mg/kg of vancomycin every 12 h and the second group 15 mg/kg every 8 h. After four doses, the peak and trough concentrations were measured from two blood samples. The primary outcome was the percentage of patients who attainted AUC more than 400. The occurrence of acute kidney injury also was evaluated after seven days.

**Results:** The mean age of patients in the every 12 h and every 8 h groups was 44.04 ± 16.55 and 42.86 ± 11.83 years, respectively. While neurosurgical issues were the most common causes of hospitalization, central nervous infections were the most common indications for vancomycin initiation. Urinary creatinine clearance was 166.94 ± 41.32 ml/min in the every 12 h group and 171.78 ± 48.56 ml/min in the every 8 h group. 46.42% of patients in the every 12 h group and 82.14% of patients in the every 8 h group attained AUC/MIC of more than 400 mg × hr/L. None of the patients in the every 12 h group reached more than 15 mcg/ml concentration. At the 7-day follow-up, 10.7% patients in the BD group and 28.6% patients in the TDS group developed acute kidney injury (*p* = 0.089).

**Conclusion:** Administration of vancomycin at a dose of 15 mg/kg every 8 h is associated with higher pharmacokinetic attainment in ARC patients. The occurrence of acute kidney injury also was not significantly higher in this therapeutic regimen. AUC/MIC monitoring is necessary in this population.

## Introduction

Vancomycin, an antibiotic with proven efficacy against Gram-positive microorganisms is widely prescribed in hospitals (Van Der Heggen et al., 2021). Incorrect dosing leads to sub-therapeutic concentrations of vancomycin, which contributes to treatment failure and antimicrobial resistance (Bakke et al., 2017). Therefore, monitoring vancomycin pharmacokinetics and therapeutic drug monitoring have gained increasing attention within the medical community. Vancomycin is eliminated through the kidneys; therefore, renal function is the most important determinant of its pharmacokinetic profile (Hirai et al., 2019; Kim et al., 2019). Altered renal function can lead to changes in vancomycin concentrations (Alzahrani et al., 2021). Augmented renal clearance (ARC) represents one such pathological condition and is defined as creatinine clearance more than 130 ml/min/1.73 m^2^ (Bilbao-Meseguer et al., 2018). ARC occurs in patients with sepsis, trauma, burns, traumatic brain injury, intracerebral hemorrhage, and major surgery (Cook and Hatton-Kolpek, 2019). Previous studies have identified young age, male sex, and illness severity as risk factors associated with the ARC phenomenon. Administration of vasopressor agents and saline loading may also increase the risk of ARC (Udy et al., 2010; Udy et al., 2013b; Luo et al., 2021). Previous studies have observed that patients with ARC have insufficient therapeutic concentrations of antibiotics; therefore, these patients are at a high risk of treatment failure. Inadequate therapeutic concentrations are more likely associated with antibiotics that undergo renal elimination (Chen and Nicolau, 2020; Zhao et al., 2022). Chu et al. conducted a retrospective study in patients receiving vancomycin at dose of 1,000 mg every 12 h. The trough of vancomycin then measured and they observed that vancomycin trough concentrations were lower than the minimum recommended range in 62.9% of patients who showed ARC. They recommended higher dose of vancomycin is needed in this population (Chu et al., 2016). Therefore, dose adjustment is necessary in such cases. Vancomycin is a time-dependent antibiotic; therefore, larger doses of this drug may be required in patients who manifest ARC (Udy et al., 2013a). To date, no clinical trial has investigated the optimal treatment regimen for management of ARC. In this clinical trial, we evaluated vancomycin pharmacokinetics after administration of two different regimens in patients with ARC.

## Materials and methods

### Design

This study was a randomized clinical trial conducted at the Loghman Hakim hospital, Shahid Beheshti University of Medical Sciences, Tehran, Iran. Patients admitted to the intensive care unit of the hospital from April 2021 to June 2022 were included in this trial.

### Study registration

This study was approved by the Research Ethics Committee of the School of Pharmacy, Shahid Beheshti University of Medical Sciences (IR.SBMU.PHARMACY.REC.1399.356). The trial also was registered in the Iranian Registry of Clinical Trials (IRCT20180802040665N1).

### Patients

The sample size of this study was estimated based on the published study by Kassel and Van Matre. (2018). The alpha and power values were considered as 0.05 and 80%. Also, 20% drop out was considered for final calculations. All patients aged over 18 years who required vancomycin therapy were assessed for the probability of ARC. The probability was checked by the ARC and ARCTIC (Augmented Renal Clearance in Trauma Intensive Care) scores (Table 1) (Barletta et al., 2017; Jabamikos et al., 2022).

**TABLE 1:** The ARC and ARCTIC scores for prediction of occurrence of ARC.

ARC score	ARCTIC score
Age <50 years: 6 scores	SrCr <0.7 mg/dl: 3 scores
Trauma: 3 Scores	Male sex: 2 scores
SOFA≤4: 1 Score	Age <56 years: 4 scores Age between 56 and 75 years: 3 scores
Interpretation	
Scores >7: High risk for ARC	Scores >6: High risk for ARC
Sensitivity	
100%	84%
Specify	
71%	68%

ARC, Augmented renal clearance; ARCTIC: Augmented Renal Clearance in Trauma Intensive Care, SOFA, Sequential Organ Failure Assessment; SrCr, Serum creatinine

Patients with high scores were evaluated for the occurrence of ARC. Eight-hour urine samples were collected from these patients, and urinary creatinine clearance evaluated. Patients whose urinary creatinine clearance was above 130 ml/min entered the randomization phase. Patients with a serum creatinine concentration more than 1.5 mg/dl, pregnant and lactating patients, and those with history of sensitivity to vancomycin were excluded from the study. Patients who received less than 4 doses of vancomycin were also excluded. Block randomization method was used with the help of an online randomization website (sealedenvelope). Fourteen blocks (four cases per block) were created. In each block, two patients were assigned to the first group and two patients to the second group. All patients signed a written informed consent form.

### Interventions

Initially, both groups received vancomycin (Exir Pharmaceutical Company, Iran) at a loading dose of 20 mg/kg. Subsequently, patients in the first group received intravenous vancomycin at a dose of 15 mg/kg every 12 h (BD group) while patients in the second group received the same dose every 8 h (TDS group). All dosing was calculated by actual body weight. The infusion rate was set to 1,000 mg per hour. One hour post-infusion, blood samples was collected to determine the peak concentration of vancomycin. Later, 30 min before administering the subsequent dose, blood samples were collected to measure the trough concentration of vancomycin. Drug concentrations were measured *via* immunoturbidimetry using Cobas Integra^®^ 400 plus (Roche Diagnostics International Ltd., Basel, Switzerland). The method of measurement is based on the kinetic interaction of microparticles in a solution. Vancomycin antibody is covalently attached to microparticles and the drug derivative is linked to a macromolecule. The kinetic interaction of microparticles in solutions, photometrically detected by turbidity. The limit of detection by this method was 1.5 mcg/ml.

### Outcomes

The demographic information of the patients was recorded, along with their clinical and paraclinical characteristics. The primary outcome was defined as the percentage of patients who attained an area under the curve (AUC)/MIC over 400. The guideline of European Committee on Antimicrobial Susceptibility Testing (EUCAST) was used for MIC simulation (Giske et al., 2022). The secondary outcome was defined as the percentage of patients who attained a trough concentration above 15 mcg/ml. The reference range for trough level was considered between 5 and 15 mcg/ml. The peak level also was considered between 20 and 40 mcg/ml (Pagana et al., 2018). The occurrence of acute kidney injury (AKI) (defined as an increase in serum creatinine of more than 0.3 mg/dl over 48 h or the development of anuria) after seven days of follow-up was also evaluated in both groups (Matuszkiewicz-Rowińska and Małyszko, 2020).

### Pharmacokinetic measures

The formula presented below was used to calculate the urinary creatinine clearance. As the urine was collected for 8 h, 480 min was considered the time parameter in the formula.

Subsequently, the constant of elimination (k), half-life of elimination, volume of distribution (V_d_), vancomycin clearance, and AUC were calculated according to the measured peak and trough values, using the formulae presented below. Using two point samples for calculation of AUC is a valid alternative for serial sampling methods (Mogle et al., 2018; Oda et al., 2021).

$$ClCr\;\left(Urinary\right)=\frac{Urine\;Cr\;\left(mg\right)\times 100}{Serum\;Cr\;\left(\frac{mg}{dL}\right)\times 480}$$

$$K=\frac{\mathrm{Ln}\;\left(\frac{Peak}{Trough}\right)}{∆T\;\left(interval\right)}$$

$$V_{d}=\frac{Dose\times \left(1-e^{-kt\left(interval\right)}\right)}{t\;\left(interval\right)\times k\times \left(C_{\max}-\left[C_{\min}\times e^{-kt\left(infusion\;time\right)}\right]\right)}$$

$$Cl\;Vancomycin=K\times V_{d}$$

$$t_{1/2\;}=\frac{0.693\times V_{d}}{Cl\;Vancomycin}$$

$$AUC=\frac{Dose\;\left(24\;hours\right)}{Cl\;Vancomycin}$$

### Statistical analysis

Fisher exact test was used for all categorical data and Student t test was utilized for all continuous data comparisons. SPSS version 21.0 (IBM Corp., Armonk, NY, United States) and GraphPad Prism 9.0 (San Diego, CA) were used for statistical analysis. *p*-value less than 0.05 was considered as significant level.

## Results

**FIGURE 1:**
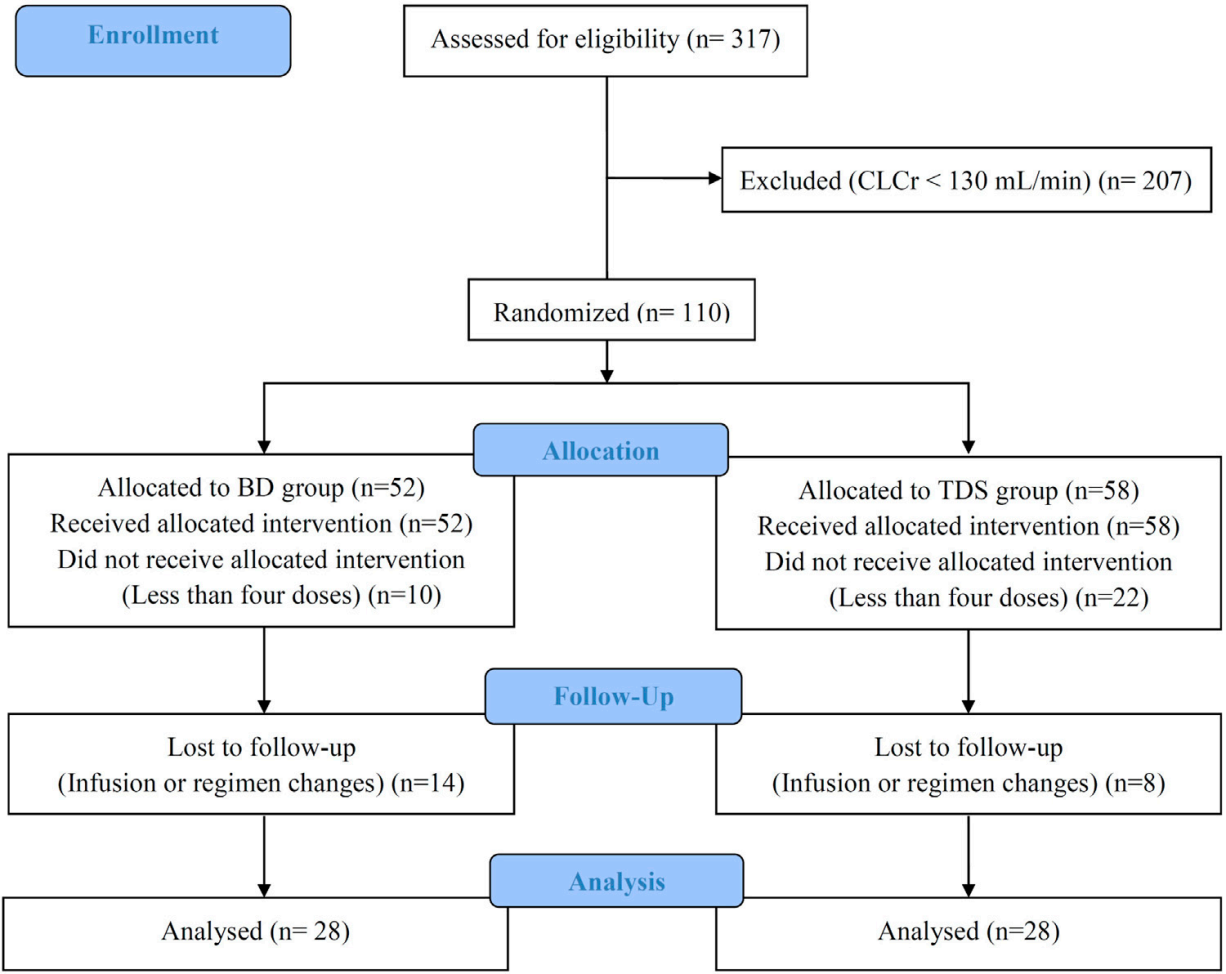
Consolidated standards of reporting trials (CONSORT) diagram of the study.

We assessed 317 patients were assessed for eligibility; after considering the inclusion and exclusion criteria, 207 were excluded, and the remaining 100 patients were randomly divided into the BD and TDS groups. Twenty eight patients in each group completed the study. The CONSORT diagram of this study is shown in Figure 1.

Seventeen (60%) patients in the BD group and 16 (57%) in the TDS group were male (*p* = 0.5). The mean age ± standard division (SD) of the patients in the BD and TDS groups were 44.04 ± 16.55 and 42.86 ± 11.83 years, respectively (*p* = 0.909). Trauma, brain tumor, intracerebral hemorrhage, and meningioma were the most frequent causes of hospitalization, and no significant differences were observed between the two groups (*p* = 0.582). The most common causes for prescribing vancomycin were meningitis, surgical prophylaxis, pneumonia, and septic shock; however, no significant differences were noted between the two groups in terms of rates of prescription (*p* = 0.783). The mean ± SD vancomycin dose in the BD and TDS groups were 1,151.79 ± 207.94 and 1,080.36 ± 118.90 mg, respectively (*p* = 0.249). The demographic details of the patient are shown in Table 2.

**TABLE 2:** Patient demographics.

		BD (n = 28)	TDS (n = 28)	*p*-Value
Gender (male) (%)		17 (60%)	16 (57%)	0.500
Age (years) (mean ± SD)		44.04 ± 16.55	42.86 ± 11.83	0.909
Diagnosis (%)	Trauma	8 (28.6%)	7 (25%)	0.582
	Brain tumor	8 (28.6%)	7 (25%)	
	Intracerebral hemorrhage	5 (17.9%)	6 (21.4%)	
	Meningioma	2 (7.1%)	5 (17.9%)	
	Others	5 (17.8%)	3 (10.7%)	
Indication of vancomycin (%)	Meningitis	11 (39.3%)	8 (28.6%)	0.783
	Surgical prophylaxis	11 (39.3%)	11 (39.3%)	
	Pneumonia	4 (14.3%)	6 (21.4%)	
	Septic shock	2 (7.1%)	3 (10.7%)	
Length of stay (days) (median)		18.5	17.5	0.421
Weight (kg) (mean ± SD)		76.07 ± 13.5	71.07 ± 9.36	0.245
Height (Cm) (mean ± SD)		171.61 ± 6.67	169.64 ± 7.81	0.303
Systolic blood pressure (mmHg) (mean ± SD)		121.4 ± 17.91	121.82 ± 16.91	0.441
Diastolic blood pressure (mmHg) (mean ± SD)		80.46 ± 13.98	79.93 ± 16.68	0.793
Heart rate (beat per minute) (mean ± SD)		78.04 ± 19.67	76.25 ± 12.66	0.863
Vancomycin dose (mg) (mean ± SD)		1,151.79 ± 207.94	1,080.36 ± 118.90	0.249

BD, Indicates every 12-hour group, TDS, Indicates every 8-hour group

The baseline serum creatinine of patients in the BD and TDS groups were 0.71 ± 0.12 and 0.62 ± 0.16 mg/dl, respectively (*p* = 0.899). After seven days, the serum creatinine in the BD and TDS groups increased to 0.92 ± 0.28 and 1.2 ± 0.39 mg/dl, respectively (*p* = 0.003). Urinary creatinine clearances in the BD and TDS group were 166.94 ± 41.32 and 171.78 ± 48.56 ml/min, respectively. The other laboratory findings were not significantly different between the two groups. The median ARC and ARCTIC scores in both groups was seven (*p* = 0.325 and *p* = 0.089). Table 3 shows the laboratory findings of both groups.

**TABLE 3:** Laboratory findings as well as ARC and ARCTIC scores of both groups.

		BD (*n* = 28)	TDS (*n* = 28)	*p*-Value
Serum creatinine (mg/dl) (mean ± SD)		0.71 ± 0.12	0.62 ± 0.16	0.899
Serum creatinine after 7 days (mg/dl) (mean ± SD)		0.92 ± 0.28	1.2 ± 0.39	0.003
8-h urine creatinine (mg) (mean ± SD)		545.5 ± 91.99	514.36 ± 128.31	0.219
Urine creatinine clearances (ml/min) (mean ± SD)		166.94 ± 41.32	171.78 ± 48.56	0.902
Platelet (×10^9^/L) (mean ± SD)		297.61 ± 118.08	318.61 ± 86.83	0.413
Hemoglobin (g/dl) (mean ± SD)		10.10 ± 1.42	10.80 ± 1.51	0.056
WBC (×10^9^/L) (mean ± SD)		10.94 ± 5.35	8.82 ± 3.44	0.156
WBC after 7 days (×10^9^/L) (mean ± SD)		9.65 ± 4.12	9.61 ± 2.84	0.688
Lactate (mg/dl) (mean ± SD)		17.79 ± 6.45	15.96 ± 3.65	0.463
Alkaline phosphatase (Units/L) (mean ± SD)		156.42 ± 58.79	139.43 ± 39.77	0.283
Alanine aminotransferase (Units/L) (mean ± SD)		28.31 ± 11.80	26.56 ± 10.49	0.755
Aspartate aminotransferase (Units/L) (mean ± SD)		42.61 ± 22.18	41.55 ± 20.20	0.889
Albumin (g/L) (mean ± SD)		2.66 ± 0.33	2.54 ± 0.36	0.198
CRP (Positive) (%)		10 (35.7%)	12 (42.9%)	0.392
Cultures (%) (From tracheal secretions and blood)	*Klebsiella pneumoniae*	3 (10.7%)	4 (14.3%)	0.794
	*Acinetobacter baumannii*	2 (7.1%)	4 (14.3%)	
	*Pseudomonas aeruginosa*	1 (3.6%)	1 (3.6%)	
	Negative	22 (78.6%)	19 (67.9%)	
ARC (median)		7	7	0.325
ARCTIC (median)		7	7	0.089

ARC, Augmented renal clearance; ARCTIC, Augmented Renal Clearance in Trauma Intensive Care; BD, Indicates every 12-hour group, CRP, C-reactive protein, TDS, Indicates every 8-hour group, WBC, White blood cells

Mean trough concentration (mean ± SD) of vancomycin was 5.64 ± 1.92 mcg/ml in the BD group and 14.03 ± 2.97 mcg/ml in the TDS group. The difference in trough concentrations between the two groups was statistically significant (*p* < 0.001). The mean peak concentrations (mean ± SD) of vancomycin in the BD and TDS groups were 20.71 ± 4.17 mcg/ml and 33.57 ± 8.34 mcg/ml, respectively; this was also a statistically significant difference (*p* < 0.001). Other pharmacokinetic parameters were calculated based on the peak and trough concentrations, constant of elimination, half-life of elimination, vancomycin clearance, and volume of distribution were not significantly different between the two groups. The AUC also was calculated in the both groups: the mean ± SD was 397.90 ± 76.02 mg × hr/L in the BD group and 611.92 ± 148.01 mg × hr/L in the TDS group (*p* < 0.001). Table 4 shows the calculated pharmacokinetic parameters in both groups.

**TABLE 4:** Calculated pharmacokinetic parameters.

	BD (*n* = 28)	TDS (*n* = 28)	*p*-Value
Trough concentration (mcg/ml) (mean ± SD)	5.64 ± 1.92	14.03 ± 2.97	<0.001
Peak concentration (mcg/ml) (mean ± SD)	20.71 ± 4.17	33.57 ± 8.34	<0.001
K (mean ± SD)	0.1411 ± 0.0339	0.1561 ± 0.0391	0.127
T _1/2_ (h) (mean ± SD)	5.19 ± 1.22	4.78 ± 1.53	0.130
Vancomycin clearances (L/hr) (mean ± SD)	5.97 ± 1.48	5.69 ± 1.87	0.306
V_d_ (L) (mean ± SD)	44.39 ± 14.21	41.87 ± 27.30	0.169
AUC (mg×hr/L) (mean ± SD)	397.90 ± 76.02	611.92 ± 148.01	<0.001

AUC, area under curve; BD, Indicates every 12-hour group; T 1/2, Half-life, TDS, Indicates every 8-hour group, Vd, volume distribution.

**FIGURE 2:**
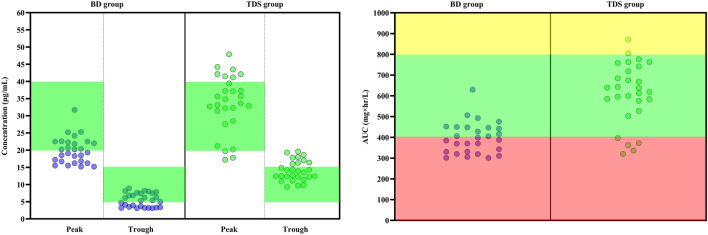
Key pharmacokinetic parameters following the administration of vancomycin with two different regimens in patients with augmented renal clearance. (Left) The peak and trough concentrations of vancomycin in the two groups. In the BD group, patients received 15 mg/kg vancomycin every 12 h and in the TDS group, patients received vancomycin at a dose of 15 mg/kg every 8 h. The green area indicates the optimal range of peak and trough levels (Trough: 5 to 15 mcg/ml and Peak: 20 to 40 mcg/ml). The green circles indicates each induvial patient of TDS group and the purple circles indicates each induvial patient of BD group. (Right) Calculated AUC/MIC is shown for both groups. The red area indicates the sub-therapeutic range of AUC (Lower than 400 mg × hr/L). The yellow area also indicates the supra-therapeutic range of AUC (Higher than 800 mg × hr/L). The green area also is the optimal range of AUC (Between 400 and 800 mg × hr/L). AUC: Area under curve, BD: Indicates every 12 h group, MIC: Minimum inhibitory concentration, TDS: Indicates every 8 h group.

According to the EUCAST database, the most commonly observed MIC for *Staphylococcus aureus* were 0.5, 1, 2, and 4 mcg/ml. The AUC/MIC was calculated for these MICs. It was hypothesized that if MIC is one of the commonly mentioned values; what will be the result. In other words, the AUC/MIC was calculated with all probable MIC values. This method is modeled from the article of Kassel and Van Matre. (2018). At an MIC of 0.5 mcg/ml, all patients achieved AUC/MIC over 400 mg×hr/L. At an MIC of 1 mcg/ml, 46.42% of patients in the BD group and 82.14% of patients in the TDS group achieved AUC/MIC over 400 mg × hr/L (*p* = 0.006). At 2 mcg/ml, none of patients in the BD group and only 7.14% of patients in the TDS group achieved the target AUC (*p* = 0.245). At an MIC of 4 mcg/ml, none of the patients in either group achieved the targeted AUC/MIC. The results also showed that none of the patients in the BD group achieved a trough concentration of more than 15 mcg/mlml and only 32.14% of patients in the TDS group achieved the same (*p* < 0.001). Figure 2 shows the results of peak concentration, trough concentration, and AUC/MIC values in both groups. At the 7-day follow-up, three (10.7%) patients in the BD group and eight (28.6%) patients in the TDS group developed AKI (*p* = 0.089). Nine (32.1%) patients in the BD and seven (25.0%) patients in the TDS group died in this study (*p* = 0.384).

## Discussion

This study was a randomized clinical trial evaluating the pharmacokinetics of vancomycin following administration of two different regimens in patients with ARC. This is the first trial that evaluated the pharmacokinetics of vancomycin, with AUC monitoring, in ARC patients. Vancomycin was administered at a dose of 15 mg/kg every 12 or 8 h in patients with creatinine clearance more than 130 mlml/min. The proportions of patients who attained a trough concentration of more than 15 mcg/mlml and AUC/MIC more than 400 were recorded. AUC/MIC was considered as the primary outcome. The AUC also should be maintained between 400 and 600 mg×hr/L. Some guidelines are also recommended to maintain the AUC below 800 mg×hr/L. These two ranges are compared in the literature and there is no definite consensus in this regard. However, we considered AUC between 400 and 800 mg×hr/L as some studies recommended it for more serious infections (Rybak et al., 2020; Tsutsuura et al., 2021; Matsumoto et al., 2022). AUC monitoring was studied as an index measure to minimize nephrotoxicity with maximal clinical effectiveness (Finch et al., 2017; Hashimoto et al., 2021). The results showed that both groups were comparable in terms of demographic characteristics and underlying conditions. The mean age of patients was 40 years; ARC is most prevalent in this age-range. The baseline laboratory findings were also similar in both groups. The results of cultures in most patients were negative. Most patients had acute central nervous system infections and negative culture results are prevalent in such patients. In addition, cultures were not performed in patients who had received vancomycin prophylaxis. The results showed that the AUC in the TDS group was significantly higher than in the BD group. He et al. performed a retrospective study to evaluate vancomycin pharmacokinetics in patients with ARC (He et al., 2020). Serum creatinine clearance was used instead of urinary creatinine clearance to diagnose ARC. Vancomycin was administered at a dose of 15.0 mg/kg every 12 h, and the area under the curve (AUC) was calculated as 232.9 ± 93.6 mg×hour/L. In the current study, the AUC in the BD group was calculated as 397.90 ± 76.02 mg×hour/L. This disparity in results may be attributable to differences in drug pharmacokinetics between the studied populations. However, the AUC in both studies showed sub-therapeutic concentrations of vancomycin in patients with ARC. He at al. did not investigate AKI occurrence. The current study also investigated the occurrence of AKI at 7-day follow-up. We observed higher AKI occurrence rates in the TDS group, although the difference was statistically nonsignificant. Notably, serum creatinine levels increased significantly after 7 days in the TDS group. In a study performed to establish an optimal vancomycin regimen in patients with various creatinine clearance rates, Vu et al. used Monte Carlo simulation and observed that patients with creatinine clearance >85 mlml/min required a vancomycin dose >2,500.0 mg/day (Vu et al., 2019). Our results concur with those of the study performed by Vu et al. Chen et al. evaluated vancomycin pharmacokinetics in patients who underwent neurosurgery (Chen et al., 2022). Based on serum creatinine clearance rates, patients were categorized into the creatinine clearance <150 ml/min and creatinine clearance >150 ml/min groups. The authors observed that only 19% of patients with serum creatinine clearance >150 ml/min attainted the target trough concentration. The mean trough concentration in the study population was 6.0 mcg/ml.

In another study by Kassel and Van Matre. (2018), the pharmacokinetics of vancomycin were evaluated in 20 neurocritically ill patients. Vancomycin was administered at a dose of 15 mg/kg (maximum 2000 mg/day) at 8- or 12-h intervals. The authors did not evaluate the patients for ARC. However, it is plausible to expect that ARC was prevalent in neurocritically ill patients. The AUC of vancomycin was 351 ± 125 mg × hr/L in the 12-hourly group and 761 ± 241 mg × hr/L in the 8-hourly group. None of the patients in the 12-hourly group attained a trough concentration of over 15 mcg/ml while 90% of patients in the 8-hourly group attained the target trough concentration. This result was similar to the current study, however target attainment was lower in the TDS group compared to the Kassel and Van Matre. (2018) study, because all patients in our study were in the ARC state. Furthermore, Kassel and Van Matre. (2018)did not evaluate the development of AKI. Although the current study evaluated the occurrence of AKI, it should be kept in mind that AKI is a multifactorial phenomenon. There may be several causes for AKI development. AKI is a syndrome that rarely has a single and distinct etiology. Many cases with AKI have a multi complex etiology where the occurrence of sepsis, ischemia and toxins often co-exist and complicate diagnosis and treatment. (Sinha Ray et al., 2016). The current study faced some limitations. The MIC was not measured because the culture results were negative. Most of the patients were neurosurgical or other surgeries cases who received antibiotics in the operation room for infection prophylaxis. This cause culture-negative result. The sensitivity of CSF culture drops by 50% if patients have already received antibiotics (Kanjilal et al., 2019). Few serum samples and limited clinical evaluation were other limitations. However, the clinical evaluation needs a more controlled setting due to its multifactorial nature.

## Conclusion

This study showed that conventional vancomycin dosing is inadequate in ARC patients. Administration of vancomycin at a dose of 15 mg/kg every 8 h (instead of every 12 h) in such patients is associated with higher pharmacokinetic attainment. However, therapeutic drug monitoring with AUC/MIC is necessary in patients with ARC.

## Data Availability

The raw data supporting the conclusion of this article will be made available by the authors, without undue reservation.
